# High-altitude pulmonary edema

**DOI:** 10.1093/emph/eoaa052

**Published:** 2021-01-06

**Authors:** Peter Woods, Joe Alcock

**Affiliations:** 1 Department of Respiratory Medicine, University Hospitals Coventry and Warwickshire, Coventry, UK; 2 Department of Emergency Medicine, University of New Mexico, Albuquerque, USA

**Keywords:** HAPE, altitude, edema, mismatch, hypoxia, vasoconstriction

## Abstract

**Lay summary:** High Altitude Pulmonary Edema (HAPE) is a potentially fatal disease of altitude, in which the lungs can become filled with fluid. In this article we explore the mechanisms causing this condition and how it can be viewed as a condition of a mismatch between current environment and evolutionary experience.

High-altitude pulmonary edema (HAPE) is a potentially lethal condition characterized by fluid accumulation in the lungs, resulting from acute exposure to high-altitude hypoxia. HAPE is a severe manifestation of high-altitude illness. This diagnostic category also includes the more common acute mountain sickness (AMS) and the more rare high-altitude cerebral edema.

HAPE is an uncommon complication of high-altitude exposure occurring after ascent above 3000 m. It is caused by two factors: increased pressure in the pulmonary circulation due to widespread pulmonary arterial vasoconstriction and an increase in capillary permeability, causing fluid to move into the alveoli in individuals susceptible to HAPE. This results in dyspnea, exercise intolerance, fatigue, cough and cyanosis. Untreated mortality approaches 50%. Treatment for HAPE includes descent, oxygen and pulmonary vasodilator medications [1]. Increasing altitude increases risk of AMS and HAPE.

Hypoxia causes widespread pulmonary vasoconstriction and capillary leak in HAPE. The cascade of inflammatory, immune and physiological changes responsible for HAPE are mediated by a variety of oxygen sensing pathways, e.g. nitric oxide synthase, vascular endothelial growth factor and hypoxia inducible factors (HIF). HIF is a key transcription factor—known as the master regulator of oxygen homeostasis [2]. It consists of a 1*α* (or 2*α*) and 1*β* subunits. Under hypoxic conditions, HIF subunits form active transcriptional complexes that stimulate expression of target genes.

## EVOLUTIONARY PERSPECTIVES

Approximately 33.5% of people live below 100 m sea level and the vast majority of humans live in locations lower than 500 m in elevation [3]. About 1% resides at altitudes above 2500 m [4]. This demographic trend is thought to be the case for human history [5]. As lowlanders increase their altitude their risk of HAPE increases.

In lowlands where most human evolution took place, hypoxia in the body occurs with infection, trauma and thromboembolism. For lowlanders, HAPE is an outcome of a mismatch between their evolved environment and the atypical environment of high altitude.

Gene-environment mismatch sheds light on HAPE vulnerability. For lowlanders with lung injury or infection, hypoxic pulmonary vasoconstriction drives blood toward healthier lung, maintaining oxygenation. This mechanism, coordinated by HIF-1, can be beneficial in localized hypoxia, as in pneumonia [6]. In HAPE, widespread hypoxia causes a dangerous rise in pulmonary blood pressure, without any benefit of shunting blood away from diseased alveoli [1]. We point out that this adaptive response in trauma or infection orchestrated by HIF-1 [7] is maladaptive at altitude.

Several populations have adaptations compensating for high-altitude hypoxia. Tibetans and Andeans have undergone selection on HIF-2*α*, leading to decreased inflammation and vasoconstriction in response to hypoxia [8]. This may confer improved exercise tolerance at high altitude and decreased incidence of HAPE [9, 10]. Whether Andeans or Tibetans suffer any tradeoffs from this is yet unknown.

## FUTURE IMPLICATIONS

Framing HAPE as gene-environment mismatch has treatment implications. Correcting mismatch by descending to lower altitude is curative. Treatments simulating descent, e.g. hyperbaric treatment or supplemental oxygen, also help. Pulmonary vasodilator drugs can help reverse pulmonary artery vasoconstriction in HAPE, however, can potentially worsen oxygenation in conditions where hypoxic pulmonary vasoconstriction has evolved such as pneumonia [11].

Are there risks to intervening in hypoxia unrelated to high altitude? Supplementary oxygen has the potential to silence HIF and downstream inflammation, possibly impeding healing, reviewed in Reference [7]. Large scale randomized trials are currently testing whether giving less supplementary oxygen might reduce mortality in critically ill patients.
